# Right Ventricular Function Improves Early After Percutaneous Mitral Valve Repair in Patients Suffering From Severe Mitral Regurgitation

**DOI:** 10.3389/fcvm.2022.830944

**Published:** 2022-03-17

**Authors:** Jonas Neuser, Hans Julian Buck, Maximiliane Oldhafer, Jan-Thorben Sieweke, Udo Bavendiek, Johann Bauersachs, Julian D. Widder, Dominik Berliner

**Affiliations:** Department of Cardiology and Angiology, Hannover Medical School, Hannover, Germany

**Keywords:** mitral regurgitation, percutaneous mitral valve repair, right ventricle, ventricular function, echocardiography, right ventricular strain

## Abstract

**Background:**

Percutaneous mitral valve edge-to-edge procedure (PMVR) using the MitraClip^®^ system (Abbot Vascular, CA) is an established therapy for severe mitral regurgitation (MR) in patients judged inoperable or at high surgical risk. Besides determining exercise capacity, right ventricular (RV) function has prognostic value in heart failure and after cardiac surgery. We therefore investigated the impact of PMVR on RV function in patients with severe MR.

**Methods and Results:**

Sixty-three patients undergoing PMVR at our department were prospectively enrolled. Transthoracic echocardiography was performed before, early (2–12d) after PMVR and after 3 months, including advanced echocardiographic analyses such as 3D imaging and strain analyses. At baseline, all patients presented with advanced heart failure symptoms. Etiology of MR was more often secondary and, if present, left ventricular (LV) dysfunction was predominantly caused by ischemic cardiomyopathy. PMVR substantially reduced MR to a grade ≤ 2 in most patients. Echocardiographic assessment revealed a largely unchanged LV systolic function early after PMVR, while in contrast RV function substantially improved after PMVR [3D RV EF (%): pre 33.7% [27.4; 39.6], post 40.0% [34.5; 46.0] (*p* < 0.01 vs. pre), 3 months 42.8% [38.3; 48.1] (*p* < 0.01 vs. pre); 2D RV GLS (%): pre −12.9% [−14.5; −10.5], post −16.0% [−17.9; −12.6] (*p* < 0.01 vs. pre), 3 months −17.2% [−21.7; −14.9] (*p* < 0.01 vs. pre)]. Factors that attenuated RV improvement were larger ventricular volumes, lower LV function, secondary MR, and a higher STS score (all *p* < 0.05).

**Conclusion:**

By using advanced echocardiographic parameters, we discovered an early improvement of RV function after PMVR that is preserved for months, independent from changes in LV function. Improvement of RV function was less pronounced in patients presenting with an advanced stage of heart failure and a higher burden of comorbidities reflected by the STS score.

## Introduction

Mitral regurgitation (MR) is the second most common valvular disease within the western world (1, 2). According to the Guidelines of the European Society of Cardiology, percutaneous mitral edge-to-edge procedure (PMVR) may be considered in patients suffering from severe MR, which are judged inoperable or at high surgical risk (2, 3). The EVEREST II trial proved that PMVR led to comparable results as conventional surgery concerning mortality or prevalence of moderate-severe or severe MR after 5 years (4). However, despite of improvement in NYHA functional class, more than 10% of patients die and almost 15% are re-hospitalized due to heart failure within the first year after PMVR using the MitraClip^®^ system (Abbott Vascular, Santa Clara, CA) (5). Parameters such as left ventricular (LV) end-systolic volume and NYHA functional class were shown to predict outcome after PMVR, but there is still a need to identify patients who may or may not benefit from PMVR and which patient require a stringent follow up (5).

Right ventricular (RV) function was shown to determine exercise capacity and to possess prognostic value for heart failure and in cardiac surgery outcome (6–12). Due to chronic volume overload, MR causes structural and hemodynamic changes, such as LV remodeling and pulmonary hypertension. These alterations in turn can lead to an increase in RV afterload causing RV remodeling and dysfunction (13–15). It has been shown that surgical therapy for MR is associated with a higher risk of postoperative RV dysfunction (16–19). There is only limited data available on the impact of MR treatment by PMVR on RV function and results are conflicting (20–23). Recent 2D echocardiographic studies reported that in contrast to surgical mitral valve repair, RV function is preserved or even slightly improved after MitraClip^®^ procedure (24–26). Beyond that, a tricuspid annular plane systolic excursion (TAPSE) <15 mm is associated with worse outcome after PMVR (27, 28). However, assessing the RV is challenging due to its complex geometric structure, retrosternal location, trabeculated endocardial surface and load dependency of function indices (25). Due to new 3D echocardiography-based methods, determination of RV volumes and function has recently become possible more easily (29).

We therefore sought to provide further insight on the influence of PMVR on ventricular function using more advanced echocardiographic methods such as 3D echocardiography and myocardial strain analysis in addition to standard 2D echocardiography in a real-world setting.

## Patients and Methods

The study protocol is in accordance with the ethical guidelines of the 1975 declaration of Helsinki and approved by the local ethic committee of Hannover Medical School (#3047-2016). All patients gave written informed consent to participate in this study. We prospectively studied consecutive patients suffering from severe MR undergoing elective PMVR using the MitraClip^®^ system at our department. In advance patients were assessed clinical, by transthoracic as well as transoesophageal echocardiography to evaluate MR severity along with mitral valve (MV) morphology. Coronary angiography was performed in all patients to exclude relevant coronary artery disease requiring revascularization. Patients were referred for PMVR by an interdisciplinary team of interventional cardiologists, cardiac imagine experts, cardiac surgeons, and cardiac anesthesiologists based on current guidelines and MV anatomy.

Patients' characteristics concerning general traits, comorbidities and laboratory values were obtained from medical records. PMVR was performed under general anesthesia and fluoroscopic as well as transoesophageal echocardiographic guidance, as described earlier (30). Preexisting co-medication was continued; so far, no preexistent or new contraindication existed. Follow-up data were obtained from medical records as well as by telephone interview 1 year after PMVR.

Sixty-three patients were initially included in the study. One patient withdrew consent for the study and one for the PMVR procedure. Fourteen patients did not attend the follow-up visit in our outpatient clinic 3 months after PMVR. During the first year, five patients deceased, while one withdrew consent for the study. Five patients were lost to follow up after 1 year, but eight patients, who did not attend the outpatient clinic for their 3 months follow-up visit, could be interviewed by phone 1 year after PMVR.

Transthoracic echocardiography using a PHILIPS EPIQ7 ultrasound machine equipped with a X5-1 transducer (PHILIPS, Amsterdam, Netherlands) was performed before and early after PMVR (2–12d) as well as 3 months after PMVR during routine follow-up in the outpatient clinic. Severity of MR was graded following the technique defined by Foster et al. (31). Images presenting the RV were recorded in standard 4-chamber view (4 CV). Analysis of RV global longitudinal strain (GLS) and fractional area change (FAC) derived from 2D images, LV GLS and global circular strain (GCS) derived from 3D images as well as biventricular 3D ejection fraction (EF) were assessed offline using TomTec Imaging Systems (Unterschleissheim, Germany).

All results are presented as median with interquartile range (IQR) or mean with standard deviation. Qualitative variables were compared using the chi^2^ test. Comparison of quantitative variables between groups were performed using the Mann-Whitney-U-Test. Changes of dependent variables over time were analyzed using a variance analysis by the Friedman method followed by Wilcoxon test in the case of significant results. *P* values were corrected for multiple testing by the Bonferroni method. Cochran's Q test was used for comparison of dependent dichotomous variables. Univariable logistic regression analysis was performed to assess predictors for RV improvement after PMVR. Multivariate analysis was not performed due to the limited number of patients in the subgroups. *P* values <0.05 were considered statistically significant. Statistical analyses were done using IBM SPSS Statistics 26 (IBM, Armonk, NY, USA).

## Results

**Figure 1:**
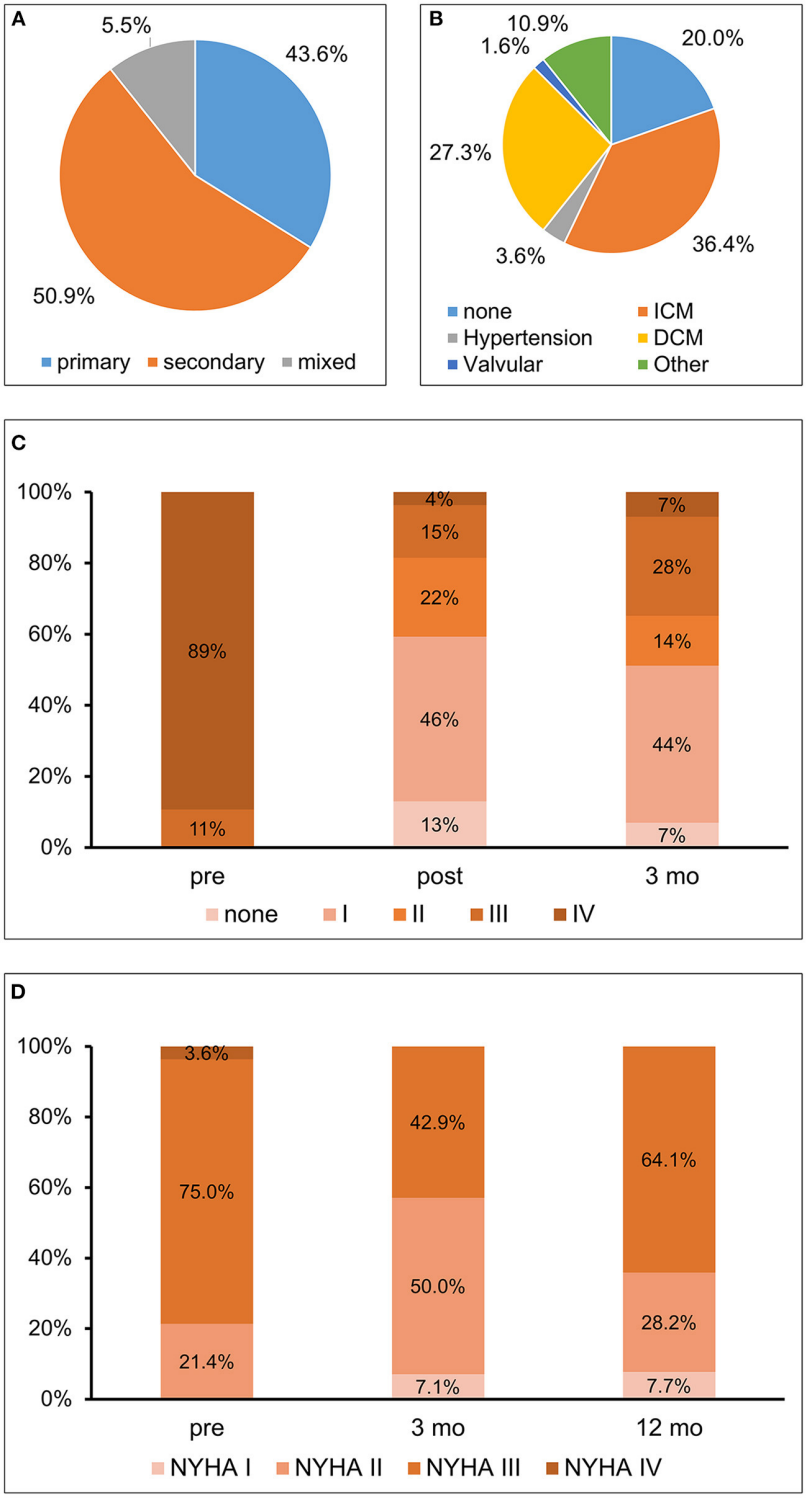
**(A)** Etiology of mitral regurgitation; **(B)** Etiology of left ventricular dysfunction; **(C)** Mitral regurgitation–Severity of mitral regurgitation at baseline (pre), post procedural, i.e., at dismission (post), and 3 months (3 mo) after percutaneous mitral valve repair (PMVR); **(D)** Functional capacity–Assessment by New York Heart Association (NYHA) class before (pre) as well as at 3 months (3 mo) and 1 year (12 mo) after PMVR. ICM, ischemic cardiomyopathy; DCM, dilative cardiomyopathy.

Most patients presented with secondary etiology of MR (Figure 1A). Median age was 80 (IQR 75-84) years and the majority of male gender (73%). Baseline characteristics of the cohort including comorbidities are depicted in Table 1. Most patients presented with a high burden of comorbidities and decompensated heart failure had occurred in almost 50%. About one third had suffered from myocardial infarction, more than half of all patients had undergone percutaneous coronary intervention, and in one third coronary bypass graft surgery had been performed. At baseline, all patients presented with symptoms of heart failure (New York Heart Association (NYHA) class ≥II). If present, LV dysfunction was predominantly caused by ischemic cardiomyopathy (Figure 1B). The majority of patients with reduced LV function received a sufficient pharmacological heart failure treatment consisting of ACE inhibitor or AT blocker (87.3%), beta-blocker (87.3%), mineralocorticoid receptor antagonists (45.5%) and diuretics (92.7%). Data on the medication during the 12 months of follow up is depicted in Supplementary Table 1. There was no significant change in the medication during the observational period. As a marker of heart failure NT-proBNP was elevated to a median of 5,356 (IQR 2028-6971) ng/l.

**Table 1:** Patient characteristics.

	**Median [IQR] or %**
**Characteristic**	
Age (years)	80 [75; 84]
Male gender	73.2%
BMI (kg/m^2^)	25.41 [23.18; 29.62]
SBP (mmHg)	122 [107; 134]
DBP (mmHg)	65 [57; 76]
Heart rate (bpm)	72 [62; 86]
EuroScore II (%)	7.04 [5.4; 12.0]
STS-Score (Mortality)	
Replacement (%)	5.54 [3.95; 7.03]
Repair (%)	5.31 [3.15; 7.39]
**Comorbidities**	
Arterial hypertension	92.9%
Diabetes mellitus	32.1%
Hyperlipidemia	73.2%
COPD	12.5%
Renal function	
GFR > 90 ml/min	1.8%
GFR 60–90 ml/min	19.6%
GFR 30–60 ml/min	57.1%
GFR 15–30 ml/min	19.6%
GFR <15 ml/min	1.8%
Atrial fibrillation	76.8%
Pacemaker	30.4%
CRT	10.7%
H/O cerebral ischemia	14.3%
H/O decompensated HF	50.0%
H/O myocardial infarction	33.9%
H/O PTCA	57.1%
H/O CABG	33.9%
**Heart rhythm**	
Sinus rhythm	30.9%
Atrial fibrillation	43.6%
Ventricular stimulation	21.8%
Other	3.6%
**Hemodynamics**	
Cardiac index (Thermo-Dilution; l/min/m^2^)	2.44 [2.24; 3.02]
Cardiac index (Fick; l/min/m^2^)	2.42 [2.09; 2.63]
Mean pulmonary arterial pressure (mmHg)	30 [23; 37
Pulmonary arterial wedge pressure (mmHg)	19 [12; 23]
Pulmonary vascular resistance (Dynes)	177 [128; 241]

*BMI, body mass index; CABG, coronary artery bypass graft; CKD, chronic kidney disease; COPD, chronic obstructive pulmonary disease; CRT, cardiac resynchronization therapy; DBP, diastolic blood pressure; GFR, glomerular filtration rate; H/O, history of; HF, heart failure; IQR, interquartile range; PTCA, percutaneous transluminal coronary angioplasty; PVR, pulmonary vascular resistance; SBP, systolic blood pressure*.

Three months after PMVR MR was reduced and most patients (58.5%) presented with a MR grade ≤ 2 (Figure 1C). No significant difference in the reduction of the MR between patients with secondary or primary MR was detectable (*p* = 0.457). Heart failure burden, evaluated by NYHA functional class, improved at 3 months, with more than one third still being NYHA I or NYHA II after 12 months (Figure 1D). During the observational period a total of nineteen hospitalizations had occurred. However, only four of them were due to cardiac decompensation. More than 50% of the events were due to non-cardiovascular causes.

**Figure 2:**
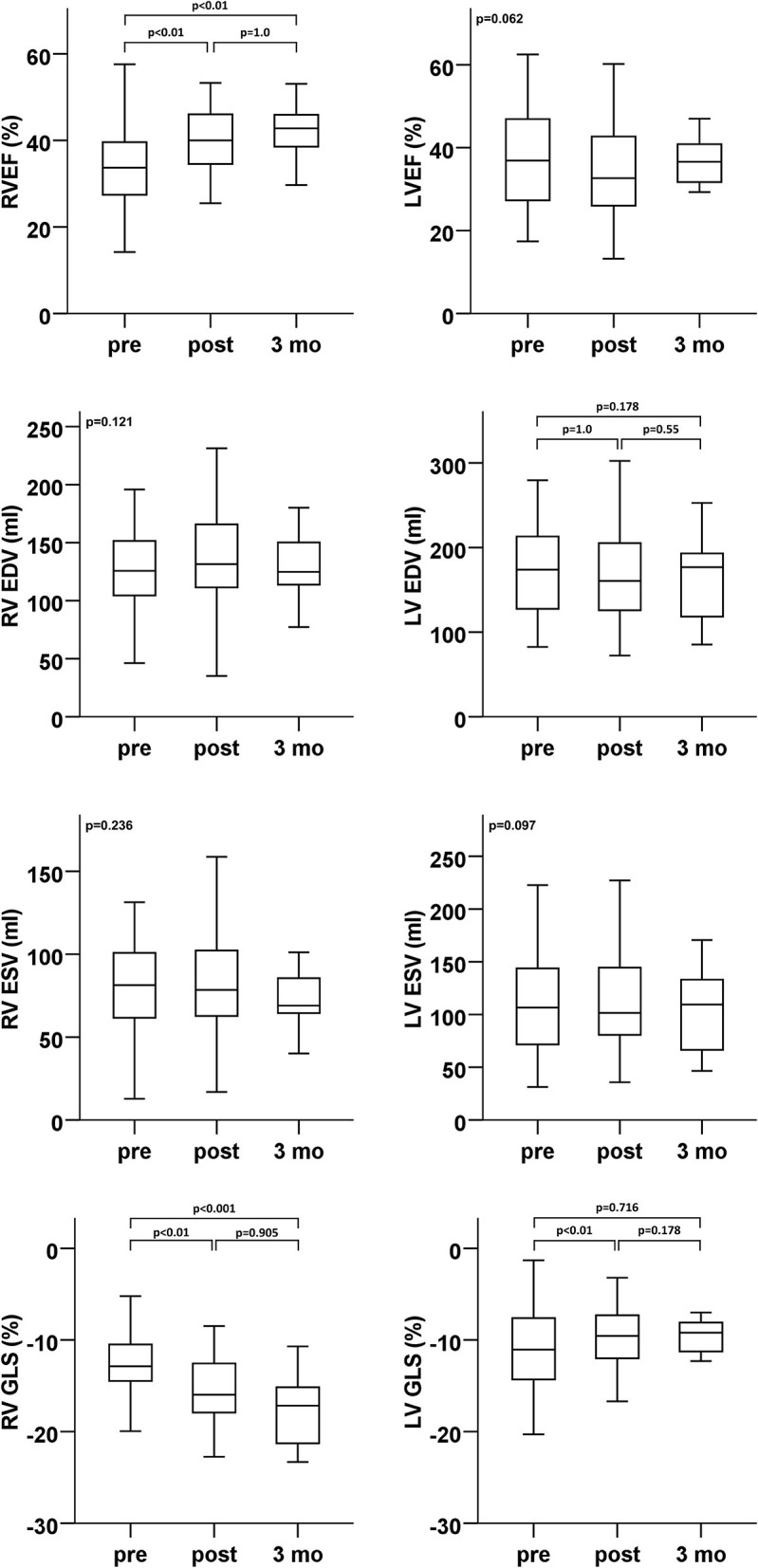
Ventricular function (ejection fraction and global longitudinal strain) and volumes (enddiastolic and endsystolic)–echocardiographic analyses of LV (right column) and RV (left column) parameters at baseline (pre), dismission (post), and 3 months after percutaneous mitral valve repair (PMVR). EDV, end diastolic volume; ESV, end systolic volume; GLS, global longitudinal strain; LV, left ventricle; LVEF, left ventricular ejection fraction; RV, right ventricle; RVEF, right ventricular ejection fraction.

Echocardiographic assessment showed only a temporary trend for a decrease in LV function (3D LVEF) early after PMVR (Figure 2 and Table 2). There were no significant changes of LV volumes (Table 2). There was a short-term deterioration of the GLS after PMVR, which was not significant at 3 months follow-up. No changes were detectable in relation to the GCS.

**Table 2:** Echocardiographic analyses of LV and RV parameters at baseline, dismission (Post PMVR), and 3 months after percutaneous mitral valve repair (Data presented as inter quartile range).

	**Baseline**	**Post PMVR**	**3 months**	***P***
**Left ventricle**				
3D LV EDV (ml)	174.0 [127.6; 213.1]	160.6 [125.9; 205.4]^n.s.^	176.9 [115.3; 197.4]^n.s.^	**0.045**
3D LV ESV (ml)	106.6 [71.7; 143.8]	101.6 [80.7; 144.5]	109.5 [64.4; 136.5]	0.097
3D LV SV (ml)	57.1 [51.1; 73.5]	55.6 [44.1; 64.6] ^n.s.^	57.1 [46.8; 61.7] ^n.s.^	**0.050**
3D LV EF (%)	36.9 [27.3; 46.9]	32.7 [26.0; 42.7]	36.6 [30.9; 41.1]	0.062
2D LV GLS (%)	−11.1 [−14.3; −7.6]	−9.6 [−12.0; −7.3]^**^	−9.2 [−11.4; −7.8]	**0.008**
3D LV GCS (%)	−15.8 [−22.1; −10.4]	−13.0 [−19.3; −11.0]	−17.3 [−19.2; −12.8]	0.236
3D LV 3D twist	8.5 [4.2; 13.3]	6.7 [2.7; 12.4]	9.9 [5.4; 13.0]	0.459
2D LVEF (Simpson bp; %)	37 [28; 51]	34 [25; 46]^**^	36 [28; 48]^*^	**<0.001**
E wave (cm/s)	103.5 [78.4; 119.0]	136.2 [93.4; 158.0]	133.8 [101.2; 153.0]	0.156
A wave (cm/s)	53.8 [33.6; 88.6]	108 [84.5; 129.0]^*^	114 [68.6; 137.6]	**0.039**
Deceleration time (ms)	170 [150; 195]	260 [220; 350]	220 [140; 340]	0.060
s‘lateral (cm/s)	5.9 [4.6; 6.6]	5.7 [4.2; 7.2]	6.1 [5.6; 6.7]	0.325
e‘lateral (cm/s)	8.25 [6.1; 10.4]	8.6 [5.6; 9.4]	7.2 [5.5; 8.4]	0.417
a‘lateral (cm/s)	5.15 [3.1; 6.3]	6.6 [3; 7.8]	6.8 [5.1; 9.1]	1.000
s‘septal (cm/s)	4.5 [4.0; 5.2]	4.1 [3.6; 5.3]	4.95 [3.9; 6.0]	0.102
e‘septal (cm/s)	4.6 [4.2; 5.8]	4.1 [3.2; 4.6]	4.3 [3.5; 5.2]	0.303
a‘septal (cm/s)	4.3 [3.4; 5.4]	3.7 [3.7; 4.8]	6 [4.4; 7.0]	0.148
E/e‘	16.6 [13.4; 21.5]	27.0 [16.2; 31.4]	25.4 [17.8; 33.5]	0.069
LA volume (ml)	132.9 [104.6; 154.9]	128.3 [97.0; 164.4]	123.15 [96.0; 174.8]	0.970
**Right ventricle**				
3D RV EDV (ml)	125.8 [101.9; 151.6]	131.5 [111.5; 165.8]	124.8 [111.1; 158.8]	0.121
3D RV ESV (ml)	81.4 [61.7; 101.1]	78.5 [62.8; 102.3]	69.0 [63.9; 90.6]	0.236
3D RV SV (ml)	42.0 [30.0; 53.7]	50.5 [43.8; 58.8]^*^	50.0 [46.0; 68.2]^**^	**0.004**
3D RV EF (%)	33.7 [27.4; 39.6]	40.0 [34.5; 46.0]^**^	42.8 [38.3; 48.1]^**^	**0.001**
2D RV GLS (%)	−12.9 [−14.5; −10.5]	−16.0 [−17.9; −12.6]^**^	−17.2 [−21.7; −14.9]^**^	**<0.001**
2D RV EDA (cm^2^)	25.9 [22.0; 30.2]	26.9 [22.7; 32.3]	23.0 [20.3; 28.1]	0.088
2D RV ESA (cm^2^)	19.6 [15.1; 24.3]	18.2 [14.2; 24.4]	15.3 [13.7; 17.9]^**^	**0.012**
2D RV FAC (%)	25 [18.4; 31.7]	31.1 [24.6; 38.5]^*^	32.5 [26.5; 40.2]^*^	**0.007**
TAPSE (mm)	16 [13; 19]	15 [14; 20]	15 [11; 18]	0.348
RV diameter (PLAX)	3.9 [3.3; 4.4]	3.95 [3.5; 4.5]	3.9 [3.4; 4.5]	0.527
RV diameter (4CV)	4.4 [3.8; 5.0]	4.5 [4.1; 5.2]	4.3 [3.8; 4.8]	0.175
RV–E wave (cm/s)	58.1 [44.0; 67.4]	59.5 [46.2; 74.1]	54.8 [45.7; 6.02]	0.905
RV–A wave (cm/s)	44.2 [31.6; 58.7]	46.1 [39.2; 53.7]	62.1 [57.0; 68.6]	0.135
RV–deceleration time (ms)	170 [130; 220]	200 [150; 260]	170 [140; 240]	0.097
RV–s‘(cm/s)	9.4 [7.7; 11.3]	10.2 [8.4; 11.8]	11.3 [8.3; 12.9]	0.103
RV–e‘(cm/s)	8.6 [6.1; 12.1]	8.3 [5.9; 10.1]	9 [5.7; 13.0]	0.584
RV–a‘(cm/s)	9.7 [4.8; 13.9]	11.3 [8.3; 14.4]	13.4 [10.0; 15.8]	0.867
RA area (cm^2^)	28.6 [24.8; 33.9]	28.6 [21.7; 33.5]	28.0 [21.7; 32.6]	0.334
Estimated sPAP (mmHg)	43.1 [37.2; 53.0]	47.7 [38.5; 57.1]	46.2 [37.7; 49.7]	0.607

*2D, 2-dimensional; 3D, 3-dimensional; 4CV, 4 chamber view; EDA, end diastolic area; EDV, end diastolic volume; EF, ejection fraction; ESA, end systolic area; ESV, end systolic volume; FAC, fractional area change; GCS, global circumferential strain; GLS, global longitudinal strain; LA, left atrium; LV, left ventricle; PLAX, parasternal long axis; RV, right ventricle; sPAP, systolic pulmonary artery pressure; SV, stroke volume; TAPSE, tricuspid annular plane systolic excursion; n.s., not significant*.

* *p < 0.05 vs. baseline*.

** *p < 0.01 vs. baseline*.

*Bold values indicate significance*.

In contrast, various RV function parameters revealed a significant improvement in RV function early after PMVR that was sustained at 3 months follow-up (Figure 2 and Table 2). 3D RVEF, 2D RV GLS, and FAC showed a significant improvement after PMVR (Table 2). However, TAPSE, as the probably most widely used RV parameter in clinical routine, did not show any significant changes. 3D calculated RV end systolic (ESV) and end diastolic volumes (ESV) did not change significantly, however, stroke volume significantly increased.

When considering different subpopulations effects on RV function seem to be more pronounced in patients suffering from primary MR. Comparing other subgroups (e.g., existence of ischemic cardiomyopathy), retrieved no significant differences in short term changes of RV function (Supplementary Tables 1A,B).

To further elucidate factors of changes in RV function and dimensions following PVMR, we further analyzed the study population by differentiating between relevant RV function improvement and lack of early improvement after PMVR. Patients with reduced RV function at baseline (*n* = 36) were divided into tertiles depending on improvement of 3D RVEF. Being in the lowest tertile (change of <5.2% RVEF) was defined as lack of RV improvement. Patients without relevant improvement of RV function had higher Society of Thoracic Surgeons (STS) scores, more frequently secondary MR, lower LV function (LVEF, GLS) and higher LV volumes, and higher RV diastolic volume with reduced longitudinal function (RV GLS). Neither pulmonary artery pressures and the pulmonary vascular resistance before PMVR nor the transmitral gradient after PMVR were shown to be different between the two groups. In summary, respective patients presented with a more advanced stage of disease (Table 3). The two groups did not differ significantly concerning factors such as age, NYHA functional class or level of NT-proBNP. Results are shown in Table 3. In univariable analysis higher LV- and RV volumes, a more restricted LV GLS, and the existence of functional MR were predictors of a lower probability of RV improvement (Table 4).

**Table 3:** Improvement vs. Non-improvement of RV function after percutaneous mitral valve repair (PMVR) in the subgroup of patients with reduced RV function (RVEF ≤ 45%) at baseline (*n* = 36).

	**Lack of relevant RV improvement** **(*n* = 10)**	**RV improvement** **(*n* = 26)**	***P***
**Age (years)**	76 [73; 80]	80 [75; 83]	0.393
**NYHA class at baseline**			0.526
I	0%	0%	
II	10.0%	23.1%	
III	90.0%	73.1%	
IV	0%	3.8%	
**NYHA class at 12 months**			0.111
I	40.0%	5.3%	
II	20.0%	26.3%	
III	40.0%	68.4%	
IV	0%	0%	
**STS-score**			
MV-Repair (mortality)	7.63 [7.3; 16.8]	4.5 [3.1; 7.2]	**0.015**
MV-Repair (morbidity & mortality)	35.7 [34.1; 36.2]	24.6 [20.7; 31.4]	**0.049**
**Secondary mitral regurgitation**	90%	44%	**0.013**
**Tricuspid regurgitation**			0.107
Grade I	11.1%	32.0%	
Grade II	22.2%	16.0%	
Grade III	0%	28.0%	
Grade IV	33.3%	8.0%	
Grade V	33.3%	16.0%	
**Mean pulmonary artery pressure (mmHg)**	30 [26; 40]	30 [26; 36]	0.823
**PVR (dyn x sec x cm** ^**−5**^ **)**	211 [151; 376]	184 [96; 238]	0.412
**Improvement of MR**			0.775
1 grade	40%	20%	
2 grades	20%	28%	
3 grades	30%	36%	
**2D LVEF (Simpson biplane; %)**	30 [24; 34]	36 [25; 51]	0.288
**3D LVEF (%)**	24.2 [19.8; 37.0]	37.6 [27.3; 47.1]	**0.039**
**3D LV EDV (ml)**	237.6 [177.6; 319.2]	163.5 [127.6]	**0.022**
**3D LV ESV (ml)**	167.2 [122.8; 259.0]	97.9 [71.7; 134.8]	**0.022**
**LV GLS (%)**	−7.3 [−9.3; −4.8]	−11.5 [14.3; −8.4]	**0.005**
**TAPSE (mm)**	15 [13; 19]	17 [13; 20]	0.869
**3D RVEF (%)**	36.3 [32.3; 39.6]	29.1 [25.8; 41.2]	**0.041**
**RV GLS (%)**	−12.96 [−14.33; −11.51]	−16.36 [−17.91; −12.20]	**0.049**
**RV FAC (%)**	24.16 [19.49; 31.3]	22.04 [13.28; 27.48]	0.201
**RV EDV (ml)**	153.4 [130.1; 195.8]	116.2 [99.7; 149.5]	**0.021**
**RV ESV (ml)**	99.3 [79.9; 131.4]	82.8 [61.7; 98.7]	0.053
**RV E (cm/s)**	74.9 [63.0; 93.8]	51.9 [41.8; 63.5]	**0.007**
**RV E-deceleration time (ms)**	225 [180; 250]	160 [120; 190]	**0.012**
**RV s' (cm/s)**	8.7 [7.8; 9.4]	10.4 [7.4; 12.1]	0.149
**RV e' (cm/s)**	8.8 [8.3; 9.1]	7.4 [5.7; 12.2]	0.071
**Estimated sPAP (mmHg)**	42 [34; 53]	40 [37; 55]	0.850
**GFR (ml/min)**	40 [31; 51]	44 [34; 56]	0.393
**Urea (mmol/l)**	15.7 [14.3; 16.7]	9.3 [6.3; 13.9]	**0.02**
**NT-proBNP (ng/l)**	5,586 [5,356; 7,714]	4,186 [1,419; 6,971]	0.714
**Max. transmitral gradient post PMVR**	10.2 [8.4; 13.4]	10.8 [8.9; 13.4]	0.788
**Mean transmitral gradient post PMVR**	3.5 [2.4; 4.0]	3.0 [2.7; 4.0]	1.000

*2D, 2-dimensional; 3D, 3-dimensional; EDV, enddiastolic volume; ESV, endsystolic volume; EF, ejection fraction; FAC, fractional area chance; GFR, glomerular filtration rate; GLS, global longitudinal strain; LV, left ventricle; MV, mitral valve; NYHA, New York Heart Association; PVR, pulmonary vascular resistance; RV, right ventricle; sPAP, systolic pulmonary artery pressure (by echocardiography); STS, Society of Thoracic Surgeons; TAPSE, tricuspid annular plane excursion*.

*Improvement of RVEF was divided into tertiles. The lowest tertile was defined as lack of relevant improvement. Bold values indicate significance*.

**Table 4:** Predictors of improvement of right ventricular function after percutaneous mitral valve repair (PMVR) in the subgroup of patients with reduced RV function (RVEF ≤ 45%) at baseline (*n* = 36) in univariable analysis.

	**OR**	**95% CI**	***P*** **-value**
STS-Score (%)–MV-Repair (mortality)	0.822	0.675–1.002	0.052
**Secondary MR**	**0.087**	**0.010–0.797**	**0.031**
3D LVEF (%)	1.074	0.994–1.161	0.072
**3D LV EDV (ml)**	**0.982**	**0.967–0.997**	**0.022**
**3D LV ESV (ml)**	**0.981**	**0.966–0.997**	**0.017**
**LV GLS (%)**	**0.685**	**0.499–0.941**	**0.019**
**3D RVEF (%)**	**0.878**	**0.777–0.993**	**0.038**
RV GLS (%)	0.939	0.754–1.171	0.577
**RV EDV (ml)**	**0.974**	**0.953–0.997**	**0.025**
**RV ESV (ml)**	**0.974**	**0.950–1.000**	**0.048**
**RV E (cm/s)**	**0.932**	**0.882–0.986**	**0.014**
**RV E-deceleration time (ms)**	**0.982**	**0.967–0.998**	**0.030**
RV e' (cm/s)	0.962	0.823–1.123	0.622
Urea (mmol/l)	0.688	0.457–1.036	0.074

*3D, 3-dimensional; EDV, enddiastolic volume; ESV, endsystolic volume; EF, ejection fraction; GLS, global longitudinal strain; LV, left ventricle; MR, mitral regurgitation; MV, mitral valve; RV, right ventricle; STS, Society of Thoracic Surgeons*.

*Bold values indicate significance*.

## Discussion

MR is the second most common valvular disease within the western world and PMVR is an increasingly used therapeutic option for patients ineligible or at high perioperative risk for conventional surgical mitral valve repair or replacement (1, 32). However, knowledge on the impact of PMVR on RV function is scarce and conflicting.

The main findings of our study are: (1) RV function improves early after PMVR using the MitraClip^®^ system. (2) This improvement is independent from changes in LV function. (3) The improvement seems to be less pronounced in patients suffering from more advanced heart disease, i.e., higher RV and LV volumes and reduced LV function and secondary MR. (4) Advanced echocardiographic methods like 3D imaging and strain analyses are superior to the standard parameter TAPSE in detecting changes in RV function.

LV function showed a transient marginal significant (only 2D LVEF and 3D LV GLS, not 3D LVEF) decrease early after PMVR. This decline was more prominent in patients with preserved or mildly reduced LVEF (>40%) due to Frank-Starling-mechanism in a volume overload state before PMVR. By reducing MR pressure load is increased, followed by a decline in EF and stroke volume. However, due to reduced volume overload EF ameliorated within the first months after PMVR. Recently, another study found similar long-term results regarding the LV in a group of patients undergoing PMVR (33). Regarding reverse remodeling of the LV involvement of the RV before PMVR (higher RV volumes, higher sPAP) seem to be existent in patient without reverse remodeling. Despite the borderline decline and following amelioration of LV function, RV function improves early after PMVR, and this development continues at 3 months. The change in RV function can be explained by reduced RV afterload after PMVR. Earlier studies report similar observations after 6 months; however, our data indicate an early improvement of RV function within the first days after PMVR, which seems to be preserved at 3 months in our study, respectively 6 months as Vitarrelli et al. describe (34).

The occurrence or presence of reduced RV function has repeatedly been associated with a worsening of patients' prognosis (35–39). For this reason, we analyzed factors that might influence reverse RV remodeling or relevant improvement of RV function. In summary, patients with less improvement of RV function were found to be in an advanced stage of their heart disease including lower LV function and higher biventricular volumes. A less impaired RV GLS was related to a greater degree of improvement of RV function. Only mildly reduced RV GLS might reflect a state of RV function in which recovery is still possible. Recently, a study in patients undergoing surgical mitral valve repair in primary MR also showed the importance of RV GLS and the prognostic impact of its short term development on myocardial recovery and rehospitalization rates (40). In a former study only patients with secondary MR were more likely to undergo reverse remodeling (41). Nevertheless, our data indicate that patients without relevant improvement in RV function more often have secondary MR and patients with primary MR have a higher increase in RVEF and RV SV in short term follow-up. In general patients suffering from secondary MR present with more comorbidities and advanced progression of their heart disease. This is reflected by our analyses revealing patients with higher STS-risk score to be less likely to develop reverse RV remodeling and by our data showing less RV improvement in patients suffering from severe LV impairment. Our data thereby suggest a threshold of LV and RV dysfunction beyond that RV recovery is unachievable. Further studies are needed to identify this threshold and evaluate whether these patients below still profit clinically from PMVR.

In our study improved RV function could be detected using advanced echocardiographic methods, while TAPSE, in turn did not reveal any significant changes. This partly is in line with earlier reports indicating TAPSE to be a less reliable parameter of RV function compared with advanced echocardiographic methods like 3D EF, RV FAC, GLS and free wall strain. However, some reports state significant changes in RV function after PVMR even measured using TAPSE (21, 42, 43), while in contrast other studies did not show any changes. For instance, in patients undergoing cardiac surgery Rong et al. reported that TAPSE in contrast to FAC, GLS and free wall strain did not predict RV dysfunction at chest closure. Grønlykke et al. described a decline in TAPSE after cardiopulmonary bypass while RV output was sustained, which was reflected in unchanged RVEF, RV GLS and FAC. This indicates that TAPSE does not reliably reflect changes in RV function (44, 45). Van Riel et al. reported that most 2D derived indices of RV function did not show any improvement of RV function after PMVR (22). However, these latter data did not encompass strain analysis, and therefore the analyzed parameters might not be sensitive enough to reliably detect changes in RV function adequately. In your study we were able to detect changes in RV function in the setting of PMVR, but only by using more advanced echocardiographic methods. This is in line with reports showing improvement in 3D RVEF after PVMR (34, 46).

The main limitation of the study is the small sample size that limits the explanatory power of subgroup analyses. Therefore, analyses of subpopulations need to be considered with precaution and require verification in a larger patient cohort. Moreover, a relevant number of patients was lost to follow up and did not attend their appointments in our outpatient clinics for the scheduled echocardiographic examination reflecting a real-world scenario. Furthermore, the follow up period was relatively short regarding ventricular remodeling. However, changes in RV parameters could be seen very early after PMVR, which suggests that long-term follow-up in this regard is neglectable. Finally, the data only derive from one center.

However, we still believe that the presented data are of significant novelty especially concerning the very early change of RV parameters and the use of advanced echocardiographic methods in the evaluation of RV function.

In summary, we could identify an improvement of RV function early after PMVR which is preserved or even pronounced at 3 months and independent from changes in LV function. Factors that reduce the potential of RV recovery after PMVR included higher LV volumes and lower LV systolic function, higher RV diastolic volume and more severely reduced RV GLS, secondary MR and a higher STS score. Our data reveal that advanced echocardiographic methods should be implemented in daily routine for evaluation of RV function since the widely used TAPSE seems to be less sensitive in reflecting RV dysfunction and its improvement and should be interpreted with caution. Further studies are needed to elucidate a threshold of LV and RV impairment beyond patients do not profit from PMVR.

## Data Availability Statement

The original contributions presented in the study are included in the article/Supplementary Material, further inquiries can be directed to the corresponding author.

## Ethics Statement

The studies involving human participants were reviewed and approved by the local Ethics Committee of Hannover Medical School (#3047-2016). The patients/participants provided their written informed consent to participate in this study.

## Author Contributions

JN, JB, JW, and DB: substantial contributions to the conception or design of the work. JN, HB, MO, J-TS, UB, and DB: the acquisition, analysis, and interpretation of data for the work. JN and DB: drafting the work. HB, MO, J-TS, UB, JB, and JW: revising it critically for important intellectual content. JN, HB, MO, J-TS, UB, JB, JW, and DB: provide approval for publication of the content and agree to be accountable for all aspects of the work in ensuring that questions related to the accuracy or integrity of any part of the work are appropriately investigated and resolved. All authors contributed to the article and approved the submitted version.

## Conflict of Interest

JN received travel support for congresses from Orion Pharma, not related to this manuscript. J-TS received travel support for congresses from Abiomed. No conflict of Interest regarding this submission. JB received honoraria for lectures/consulting from Novartis, Vifor, Bayer, Servier, Abiomed, Pfizer, Boehringer Ingelheim, AstraZeneca, Cardior, Daichii Sankyo, CVRx, BMS, MSD, Amgen, Corvia, not related to this article, and research support for the department from Zoll, CVRx, Vifor, Abiomed, not related to this article. JW is a consultant for Biosensor/NVT and Medtronic and reports personal fees from Edwards, Daiichi Sankyo, Biotronik, Volcano/Philips all outside the submitted work. DB received honoraria or travel support from Abbott, Bayer, Biotronik, Boehringer Ingelheim, Daiichi Sankyo, Novartis, and Orion Pharma, and research support from CVRx, Novartis, and Zoll, all not related to this manuscript. The remaining authors declare that the research was conducted in the absence of any commercial or financial relationships that could be construed as a potential conflict of interest.

## Publisher's Note

All claims expressed in this article are solely those of the authors and do not necessarily represent those of their affiliated organizations, or those of the publisher, the editors and the reviewers. Any product that may be evaluated in this article, or claim that may be made by its manufacturer, is not guaranteed or endorsed by the publisher.
