# Supplementary material for: Right Ventricular Function Improves Early After Percutaneous Mitral Valve Repair in Patients Suffering From Severe Mitral Regurgitation
Source: Front Cardiovasc Med. 2022 Mar 17;9:830944. doi: 10.3389/fcvm.2022.830944 (PMC8968125; doi:10.3389/fcvm.2022.830944)

Supplementary Material

Suppl. table 1: Overview of medication throughout the observational period.

| Drug class | Baseline | 3 months after PMVR | 12 months after PMVR | p |
| --- | --- | --- | --- | --- |
| ACEi/ARB/ARNI | 49/56 (87.5%) | 38/42 (90.5%) | 30/38 (78.9%) | 0.121 |
| BB | 49/56 (87.5%) | 35/42 (83.3%) | 31/38 (81.6%) | 0.472 |
| MRA | 26/56 (46.4%) | 26/42 (61.9%) | 21/38 (55.3%) | 0.236 |
| Digitalis | 10/56 (17.9%) | 9/42 (21.4%) | 6/38 (15.8%) | 0.717 |
| Loop diuretics | 51/56 (91.1%) | 38/42 (90.5%) | 35/38 (92.1%) | 0.779 |
| Other diuretics | 10/56 (17.9%) | 7/42 (16.7%) | 4/38 (10.5%) | 0.180 |

ACEi – angiotensin converting enzyme inhibitor, ARB - angiotensin receptor blocker, ARNI – angiotensin receptor-neprilysin inhibitor, BB – beta blocker, MRA – mineralocorticoid receptor antagonist, PMVR - percutaneous mitral valve repair

**Supplemental table 2A: Overview in differences of the echocardiographic parameters under different clinical conditions** (existence of ischemic cardiomyopathy, existence of reduced right ventricular function at baseline (TAPSE ≤ 16 mm), and existence left ventricular dysfunction (left ventricular ejection fraction ≤ 40%).

|  | Ischemic cardiomyopathy | | | TAPSE | | | Left ventricular function (3D LVEF) | | |
| --- | --- | --- | --- | --- | --- | --- | --- | --- | --- |
|  | no | yes | p | ≤ 16 mm | > 16 mm | p | ≤ 40% | > 40% | p |
| Δ 3D LV EDV (ml) | -10.6 [-21.1; 1.5] | 17.2 [-7.8; 38.5] | **0.008** | -10.9 [-21.1; 1.5] | -3.6 [-17.8; 18.8] | 0.510 | -6.8 [-19.5; 21.1] | -4.4 [-21.6; 6.3] | 0.630 |
| Δ 3D LV ESV (ml) | -1.3 [-12.2; 11.7] | 12.5 [-9.75; 38.7] | 0.114 | -2.2 [-10.9; 14.5] | 0.4 [-16.6; 10.8] | 0.790 | -6.0 [-15.1; 20.5] | 6.1 [-1.3; 13.6] | 0.455 |
| Δ 3D LV SV (ml) | -9.7 [-14.6; 1.2] | 0 [-9.4; 9.6] | 0.114 | -10.6 [-14.7; -0.6] | -4.9 [-14.2; 8.8] | 0.245 | -1.4 [-10.6; 9.1] | -13.1 [-20.8; -2.2] | **0.033** |
| Δ 3D LV EF (%) | -3.8 [-7.2; -0.4] | -2.4 [-6.1; 3.7] | 0.588 | -3.8 [-6.6; -1.2] | -2.3 [-7.5; 6.3] | 0.292 | -1.7 [-4.8; 5.1] | -6.2 [-9.8; -3.6] | **0.006** |
| Δ 3D LV GLS (%) | 0.8 [-2.1; 3.9] | 1.0 [-0.7; 3.7] | 0.865 | 0.9 [0.5; 3.3] | 0.6 [-2.5; 2.8] | 0.363 | 0.3 [-3.1; 2.1] | 2.5 [0.7; 4.3] | **0.009** |
| Δ 3D LV GCS (%) | 2.4 [-0.9; 4.3] | 1.1 [-0.6; 2.7] | 0.312 | 2.5 [-0.6; 3.2] | 1.4 [-2.2; 4.3] | 0.510 | 1.2 [-2.4; 2.4] | 3.9 [0.3; 5.85] | **0.009** |
| Δ 2D-EF (%) | -2 [-7; 2] | -3 [-6; -1] | 0.316 | -4 [-7; 0] | -2 [-6; 0] | 0.732 | -1 [-6; 2] | -3 [-7; 1] | 0.647 |
| Δ 3D RV EDV (ml) | 8.5 [-9.4; 25.5] | 18.4 [-13.5; 37.0] | 0.607 | 15.3 [-2.5; 22.8] | 6.9 [-12.9; 53.7] | 0.878 | 17.2 [-9.4; 43.8] | 7.4 [-11.2; 17.1] | 0.225 |
| Δ 3D RV ESV (ml) | -7.1 [-13.9; 5.5] | 0 [-8.0; 24.1] | 0.546 | -1.2 [-10.6; 5.5] | -5.5 [-18.0; 31.0] | 0.851 | 0.9 [-22.3; 24.5] | -6.6 [-13.9; 3.2] | 0.400 |
| Δ 3D RV SV (ml) | 11.6 [4.6; 19.0] | 12.7 [8.9; 22.8] | 0.872 | 12.8 [8.9; 16.9] | 12.8 [0.7; 37.1] | 0.851 | 13.8 [8.9; 27.4] | 9.2 [1.8; 19.0] | 0.304 |
| Δ 3D RV EF (%) | 7.5 [4.6; 10.9] | 6.5 [4.2; 11.9] | 0.648 | 6.4 [2.8; 11.9] | 9.7 [5.9; 11.0] | 0.297 | 7.1 [4.6; 13.1] | 8.7 [6.3; 11.5] | 0.658 |
| Δ sPAP (mmHg) | 7.5 [-4.5; 17.7] | 2.55 [-10.2; 15.1] | 0.571 | -0.7 [-8.4; 12.3] | 15.5 [-9.5; 29.1] | 0.404 | -5.3 [-9.5; 23.9] | 5.8 [-0.7; 8.0] | 0.620 |
| Δ RV-GLS (%) | -2.8 [-4.7; -0.9] | -3.4 [-5.4; -1.3] | 0.867 | -3.3 [-4.7; -1.4] | -2.8 [-4.6; -1.3] | 0.660 | -2.5 [-4.6; -0.9] | -3.3 [-5.4; 1.9] | 0.922 |
| Δ RV EDA (cm²) | 0.7 [-3.6; 4.5] | 0.3 [-1.5; 5.9] | 0.389 | 1.6 [-1.8; 5.1] | 0.3 [-2.6; 6.1] | 0.970 | 0.3 [-2.9; 4.6] | 2.5 [-1.8; 6.2] | 0.261 |
| Δ RV ESA (cm²) | -1.4 [-4.9; 1.0] | -0.3 [-2.0; 5.4] | 0.064 | -1.1 [-5.2; 3.2] | -0.3 [-2.7; 1.9] | 0.571 | -1.2 [-5.1; 1.6] | -0.5 [-1.5; 3.3] | 0.240 |
| Δ FAC (%) | 6.7 [2.5; 11.8] | 1.3 [-5.2; 5.1] | **0.011** | 6.5 [3.3; 9.6] | 5.1 [0.3; 11.2] | 0.792 | 4.8 [0.5; 10.8] | 5.8 [-0.3; 12.3] | 0.942 |
| Δ TAPSE (mm) | 0 [-2; 4] | 1 [-2; 5] | 0.615 | 1 [-1; 5] | -2 [-3; 3] | 0.074 | 1 [-2; 5] | -1 [-2; 2] | 0.422 |
| Δ RV-s‘ (cm/s) | -0.3 [-1.6; 1.0] | 2.5 [-0.8; 3.8] | **0.012** | 0.3 [-1.0; 1.4] | -0.1 [-1.6; 1.8] | 0.856 | 0.5 [-0.6; 2.5] | 0.3 [-1.9; 1.0] | 0.218 |

2D - 2-dimensional; 3D - 3-dimensional; EDA - end diastolic area; EDV - end diastolic volume; EF - ejection fraction; ESA - end systolic area; ESV - end systolic volume; FAC - fractional area change; GCS - global circumferential strain; GLS - global longitudinal strain; LV - left ventricle; RV - right ventricle; sPAP - systolic pulmonary artery pressure; SV - stroke volume; TAPSE - tricuspid annular plane systolic excursion.

**Supplemental table 2B: Overview in differences of the echocardiographic parameters under different clinical conditions** (etiology of mitral regurgitation, and success of percutaneous mitral valve repair (degree of improvement).

|  | Mitral regurgitation | | | Improvement of MR | | |
| --- | --- | --- | --- | --- | --- | --- |
|  | Primary or mixed | secondary | p | <2 grades | ≥2 grades | p |
| Δ 3D LV EDV (ml) | -4.0 [-14.0; 10.6] | -8.8 [-20.9; 13.0] | 0.641 | 2.8 [-19.5; 32.3] | -6.8 [-17.0; 13.0] | 0.433 |
| Δ 3D LV ESV (ml) | 4.2 [-4.4; 15.5] | -7.0 [-15.9; 16.9] | 0.326 | 8.0 [-4.4; 15.5] | 0.4 [-13.7; 19.9] | 0.392 |
| Δ 3D LV SV (ml) | -8.5 [-14.6; 7.0] | -6.7 [-13.9; 5.2] | 0.940 | -2.1 [-20.3; -0.6] | -7.7 [-13.9; 8.1] | 0.566 |
| Δ 3D LV EF (%) | -3.8 [-8.0; -2.3] | -3.7 [-7.0; 2.3] | 0.577 | -3.8 [-11.7; 2.2] | -3.4 [-7.0; 0.9] | 0.519 |
| Δ 3D LV GLS (%) | 1.8 [0.4; 4.2] | 0.7 [-2.9; 2.9] | 0.198 | 2.4 [0.9; 4.0] | 0.7 [-1.8; 3.6] | 0.176 |
| Δ 3D LV GCS (%) | 2.3 [-0.9; 4.3] | 1.4 [-0.6; 3.25] | 0.537 | 1.4 [-0.7; 3.0] | 2.4 [-0.8; 3.9] | 0.848 |
| Δ 2D-EF (%) | -3 [-9; 1] | -2 [-5; 0] | 0.508 | -3 [-12; -1] | -2 [-6; 1] | 0.181 |
| Δ 3D RV EDV (ml) | 14.0 [-0.4; 35.5] | 0.5 [-13.5; 20.6] | 0.191 | 11.5 [-2.5; 37.0] | 11.3 [-9.9; 25.6] | 0.807 |
| Δ 3D RV ESV (ml) | -2.3 [-12.5; 25.3] | -7.1 [-22.4; 5.5] | 0.223 | -1.3 [-10.6; 37.4] | -4.8 [-13.2; 6.0] | 0.590 |
| Δ 3D RV SV (ml) | 17.1 [8.3; 39.2] | 9.6 [-0.5; 14.1] | **0.036** | 11.0 [8.1; 21.4] | 13.2 [5.5; 20.9] | 0.832 |
| Δ 3D RV EF (%) | 9.7 [6.7; 14.1] | 5.2 [3.8; 10.2] | **0.029** | 5.8 [4.2; 6.5] | 9.1 [4.6; 11.7] | 0.286 |
| Δ sPAP (mmHg) | 4.2 [-2.2; 7.5] | 14.0 [-12; 24.8] | 0.573 | 11.6 [-6.4; 26.9] | 6 [-6.9; 14.0] | 0.617 |
| Δ RV-GLS (%) | -3.3 [-6.0; 0.6] | -2.5 [-3.9; -1.4] | 0.684 | -3.3 [-6.2; 1.3] | -2.8 [-5.3; -1.4] | 0.950 |
| Δ RV EDA (cm²) | 2.0 [-1.7; 6.2] | -0.7 [-5.1; 3.3] | **0.049** | 1.6 [-1.7; 7.6] | 0.3 [-2.5; 5.0] | 0.360 |
| Δ RV ESA (cm²) | -0.5 [-1.5; 3.2] | -2.5 [-6.2; 1.3] | 0.052 | 0.4 [-2.9; 7.0] | -1 [-3.5; 1.3] | 0.309 |
| Δ FAC (%) | 5.2 [0.3; 11.2] | 5.6 [0.5; 10.3] | 0.748 | 4.5 [-6.0; 10.0] | 4.8 [0.4; 10.3] | 0.556 |
| Δ TAPSE (mm) | -1 [-3; 1] | 2 [-1; 5] | 0.143 | -2 [-2; 1] | 2 [-2; 5] | 0.178 |
| Δ RV-s‘ (cm/s) | -0.3 [-2.2; 1.0] | 1.0 [-0.8; 2.9] | 0.078 | 2.3 [0.3; 3.8] | -0.3 [-1.6; 1.2] | **0.016** |

2D - 2-dimensional; 3D - 3-dimensional; EDA - end diastolic area; EDV - end diastolic volume; EF - ejection fraction; ESA - end systolic area; ESV - end systolic volume; FAC - fractional area change; GCS - global circumferential strain; GLS - global longitudinal strain; LV - left ventricle; MR - mitral regurgitation; RV - right ventricle; sPAP - systolic pulmonary artery pressure; SV - stroke volume; TAPSE - tricuspid annular plane systolic excursion.

**Supplemental Figure 1 – Consort flow diagram of the study –** echocardiography was performed before and early after percutaneous mitral valve repair (PMVR) as well as 3 months after PMVR during routine follow-up in the outpatient clinic; follow up after 12 months was an interview by phone (FU – follow up, PMVR - percutaneous mitral valve repair)


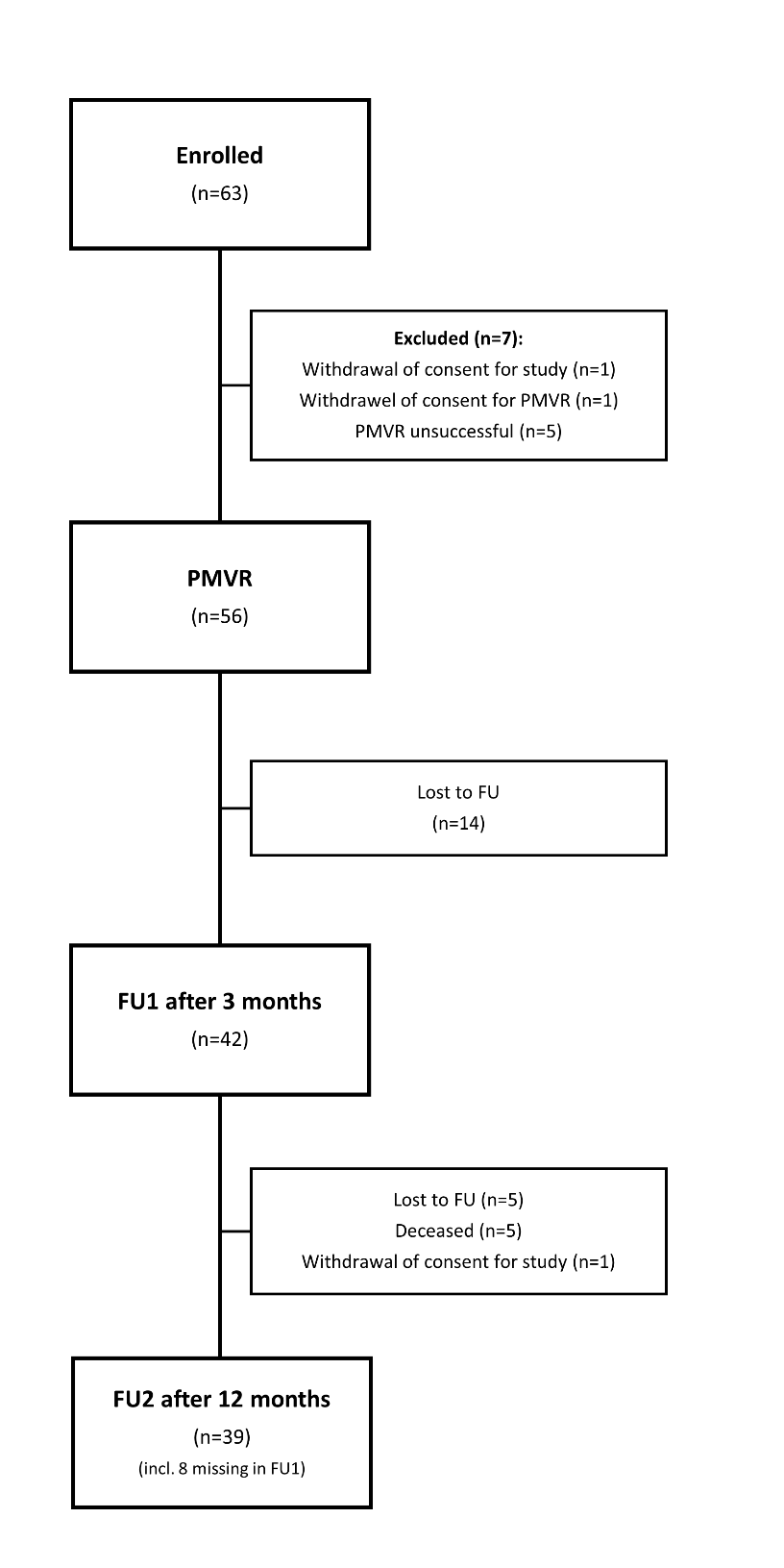

Supplement: Supplementary file 1 [file Data_Sheet_1.docx]
